# Twenty-year Follow-up of Patients With Epidemic Glomerulonephritis due to *Streptococcus zooepidemicus* in Brazil

**DOI:** 10.1016/j.ekir.2022.06.011

**Published:** 2022-06-30

**Authors:** Sergio Wyton Pinto, Helbert do Nascimento Lima, Thalles Trindade de Abreu, Alba Otoni, Paulo Cesar Koch Nogueira, Ricardo Sesso

**Affiliations:** 1 Division of Nephrology, Hospital São João de Deus, Divinopolis, Minas Gerais, Brazil; 2 Department of Medicine, Universidade da Região de Joinville, Joinville, Santa Catarina, Brazil; 3 Federal University of São João del Rei, Divinópolis, Minas Gerais, Brazil; 4 Department of Pediatrics, Federal University of São Paulo, São Paulo, Brazil; 5 Division of Nephrology, Federal University of São Paulo, São Paulo, Brazil

**Keywords:** acute nephritis, epidemic nephritis, follow-up, post-streptococcal glomerulonephritis, *Streptococcus zooepidemicus*

## Abstract

**Introduction:**

Post-streptococcal glomerulonephritis (PSGN) has a good prognosis in children, but few studies have evaluated the long-term renal outcomes in adults with PSGN.

**Methods:**

In a follow-up study, 47 predominantly adult patients with PSGN due to group C *Streptococcus zooepidemicus* were reassessed 20 years after an outbreak in Nova Serrana, Brazil. We evaluated clinical characteristics, renal outcomes, and the trajectory of the estimated glomerular filtration rate (eGFR) by the creatinine-based chronic kidney disease-epidemiology collaboration equation from 5 follow-up assessments. Logistic regression and mixed-effects regression were used in the analysis.

**Results:**

After 20 years, the participants’ mean age was 56.6±15.1 years. Thirty-four (72%) patients had hypertension, 21 (44.7%) had eGFR <60 ml/min per 1.73 m^2^, 8 of 43 (18.6%) had urine protein-to-creatinine ratio >150 mg/g, and 25 (53%) had CKD (low eGFR and/or increased proteinuria). Increasing age was associated with CKD (odds ratio: 1.07; 95% confidence interval [CI]: 1.02–1.13; *P* = 0.011) in multivariate analysis. The mean eGFR decline in the last 11 years of follow-up was −3.2 ml/min per 1.73 m^2^ per year (95% CI: −3.7 to −2.7). Older age at baseline (coefficient −1.05 ml/min per 1.73 m^2^ per year; 95% CI −1.28 to −0.81; *P* < 0.001), and hypertension 5 years after the outbreak (coefficient −7.78 ml/min/1.73 m^2^; 95% CI −14.67 to −0.78; *P* = 0.027) were associated with lower eGFR during the whole study period.

**Conclusion:**

There was a marked worsening of renal function and a high prevalence of CKD and hypertension after 20 years of PSGN outbreak. Long-term follow-up is warranted after PSGN, especially among older patients.

## Graphical abstract

**Figure:**
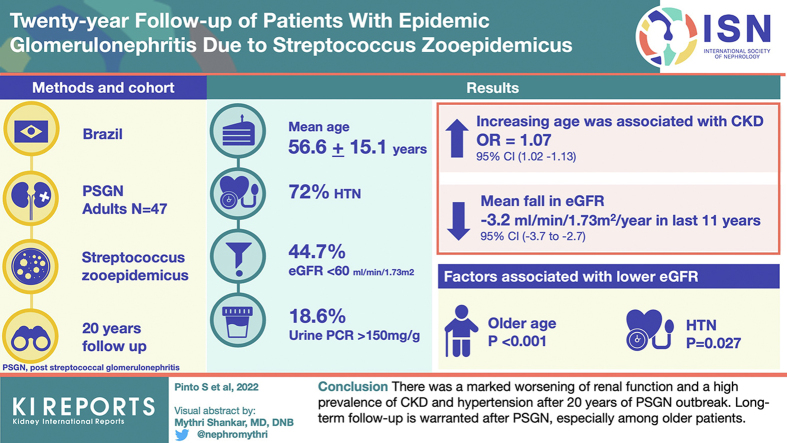

Outbreaks of PSGN are rare in adults, and few studies have evaluated patients’ long-term renal function after contracting the disease.^1^, ^2^, ^3^ Therefore, we followed up with patients affected by a large glomerulonephritis (GN) epidemic associated with Lancefield group C *Streptococcus zooepidemicus* in Minas Gerais, Brazil since 1998.^3^, ^4^, ^5^, ^6^

In 1998, 152 patients presented with fever, headache, and myalgia, followed by cervical adenopathy in a rural region near the city of Nova Serrana, in Minas Gerais, the second most populated Brazilian state. Then, 134 patients developed GN 7 to 10 days after the infection.^4^ Histologic examination of renal biopsy samples of patients with worse clinical presentation showed a postinfectious GN pattern, with varying severity, proliferative and crescentic glomerular disease.^4^ The GN outbreak was attributed to consuming unpasteurized cheese contaminated with *S zooepidemicus.*^4^ Most (90%) patients were adults, 72% were hospitalized, 98% had edema, all had hematuria, 58% had serum creatinine levels greater than 1.2 mg/dl, and 7.5% needed acute hemodialysis.^5^ Two years after the acute episode, 42% of subjects had hypertension, 12% had serum creatinine levels greater than 1.2 mg/dl, and 34% had increased microalbuminuria.^5^ Five years after the acute episode, 5 patients (3.7% of the original sample) required chronic hemodialysis.^6^ Although the proportion of patients with hypertension and microalbuminuria had decreased, there were more patients with reduced renal function over the 5 years.^6^ An improvement of the eGFR was observed 10 years after the outbreak, with mean levels a little more than those after 2 years of the disease onset, and associated with a decreased proportion of patients with albuminuria and increased hypertension rates.^3^

Although PSGN has a good prognosis in children,^1^^,^^7^, ^8^, ^9^, ^10^, ^11^ few studies have focused on the long-term outcome in adult patients.^6^^,^^12^^,^^13^ PSGN following epidemic infections in adults seems to be associated with a higher risk of chronic renal complications.^3^^,^^6^^,^^14^^,^^15^ Nevertheless, few studies with ample follow-up time have focused on PSGN in adult patients with nongroup A streptococcal strain.^16^^,^^17^ This study describes the follow-up of patients with PSGN due to *S zooepidemicus* 20 years after the acute episode.

## Methods

### Study Design and Sampling

This follow-up study consisted of 2 analysis subsets as follows: (i) a cross-sectional analysis of patients initially affected during the PSGN outbreak in 1998 and recontacted for a new clinical and laboratory evaluation in 2019; and (ii) a retrospective cohort analysis of the eGFR trajectory for all patients with serum creatinine values available in the last evaluation (2019) and taking into account their creatinine values in the 4 previous evaluations (1998, 2000, 2003, and 2008). The local nephrology team saved the medical record data of the affected patients with their contact addresses with 1 of the authors of this article (SWP). Therefore, 47 subjects were found for reevaluation 20 years after the outbreak, in the current study. The serum creatinine-based eGFR available from previous surveys of these 47 patients was used to assess their evolution.

A total of 253 cases of GN were reported in the state of Minas Gerais, Brazil, from December 1997 to July 1998. Most (92%) patients resided in Nova Serrana and Quilombo da Gaia (a small village in the city of São Gonçalo do Pará, 5%), both small towns in the center-west region of the state (populations of 27,500 and 12,000, respectively). The complete description of the outbreak has already been published.^4^ First, GN was defined by at least 3 symptoms, namely systolic blood pressure higher than 140 mmHg or diastolic blood pressure higher than 90 mmHg, edema, and at least trace hematuria or 30 mg/dl proteinuria in a previously healthy patient. Therefore, 134 patients presented with criteria for GN, and their findings in prior surveys have been previously published.^3^, ^4^, ^5^, ^6^ Next, a new clinical survey was conducted from July to September 2019 (20 years after the outbreak). After confirming their addresses in the local medical files, a research team member (SWP) contacted the original GN patients. All contacted patients were invited for a home clinical reassessment by clinical examination and collection of laboratory tests. The participants signed written informed consent. The Ethics Committee of the Federal University of São Paulo approved this study.

### Procedures and Variables Collected

The medical team visited the participants in their homes, collected the clinical information, obtained early morning blood (drawn after fasting), and recently voided urine samples for analysis. An interim history was obtained, and a physical examination was performed.

The study variables included gender, age, comorbidities (diabetes, obesity, and systemic arterial hypertension [SAH]), laboratory values (total cholesterol, high-density lipoprotein cholesterol, low-density lipoprotein cholesterol, triglycerides, hematocrit, hemoglobin, uric acid, glycated hemoglobin, glucose, and proteinuria), and the use of angiotensin-converting enzyme inhibitors or angiotensin II receptor blockers. Blood pressure was measured with a mercury sphygmomanometer while the patient was sitting after 5 minutes of rest. The average of 3 measurements taken with 1-minute intervals was used in the analysis. Hypertension was defined as systolic blood pressure was higher than or equal to 140 mmHg, or diastolic blood pressure was higher than or equal to 90 mmHg (or use of antihypertensive medication). Diabetes was defined as glycated hemoglobin was higher than 7% or by hypoglycemic drug use. The creatinine-based chronic kidney disease-epidemiology collaboration equation, without the race correction, was used to estimate eGFR.^18^ We observed eGFR evolution among the participants enrolled in the last visit (2019) and used their available serum creatinine results in the previous assessments since the outbreak. Proteinuria measurements (24-hour albuminuria at the 2003 visit and protein-to-creatinine ratio at the 2019 visit) and hypertension status (present or not) were obtained in the 2003 and 2019 follow-up assessments.

### Laboratory Analyses

Blood and urine laboratory analysis was conducted in the same laboratory using the same methodology as in all surveys performed since 1998. Blood and urine samples were appropriately stored and transported to the reference laboratory at 4°C. When examined within 1 day, urine samples were kept at 4°C; otherwise, they were frozen at −20°C. In all surveys performed since 1998, blood samples were analyzed for serum creatinine (alkaline picrate method) using a spectrophotometer. Albuminuria values were assessed by radioimmunoassay using gamma counter equipment (Gamma C12; Diagnostic Products Corporation, Los Angeles, CA) in 2003, and the values were considered abnormal if greater than 20 mg per 24 hours. Urine protein was tested in 2019 using a colorimetric assay kit (Gold Análise Diagnóstico LTDA, Minas Gerais, Brazil) and expressed as protein-to-creatinine ratio; the values were considered abnormal if greater than 150 mg/g.

### Statistical Analysis

The χ2 test was used for the comparison of categorical variables. *t*-test or the Mann-Whitney test (whenever appropriate) were used to compare continuous variables. We evaluated the variables associated with CKD by logistic regression analysis; odds ratios and 95% CI were calculated. CKD was defined as eGFR less than 60 ml/min per 1.73 m^2^ and/or the presence of proteinuria equal to or greater than 150 mg/g. All clinically plausible variables with a *P*-value lower than 0.20 to the outcome in the univariate method were included in the multivariate logistic regression analysis. After using a manual backward elimination, a final multivariate model was proposed to adjust for all significant variables in the model. The average eGFR reduction was calculated by subtracting the eGFR in 2019 from the 2008 value, and then dividing by the number of years in the range for patients with measurements in both evaluations. We evaluated the eGFR trajectory over the 20-year follow-up in the available sample at the last survey (*n* = 47) by mixed-effect linear regression analysis, taking into account their measurements in the 1998, 2000, 2003, 2008, and 2019 surveys, with the eGFR being the outcome of this analysis. The random effect was the subject identification variable. The other covariates were fixed (follow-up time, gender, age at baseline, presence of hypertension, and albuminuria [higher than 20 mg/24h] in 2003). Interaction terms between covariates and time were assessed in the models.

We considered the presence of hypertension and albuminuria values from the 2003 assessment in the analysis because that was the visit with the most data available. Similarly, all variables associated with the outcome in the univariate method with a *P*-value lower than 0.20 were included in the multivariate linear mixed-effect model analysis. A final multivariate model was proposed after using a manual backward elimination to adjust for all significant variables in the model. Tests were 2-sided and statistical significance was set at *P* < 0.05. All data were analyzed using STATA/IC 16.1 software program (StataCorp, College Station, TX USA).

## Results

**Figure 1:**
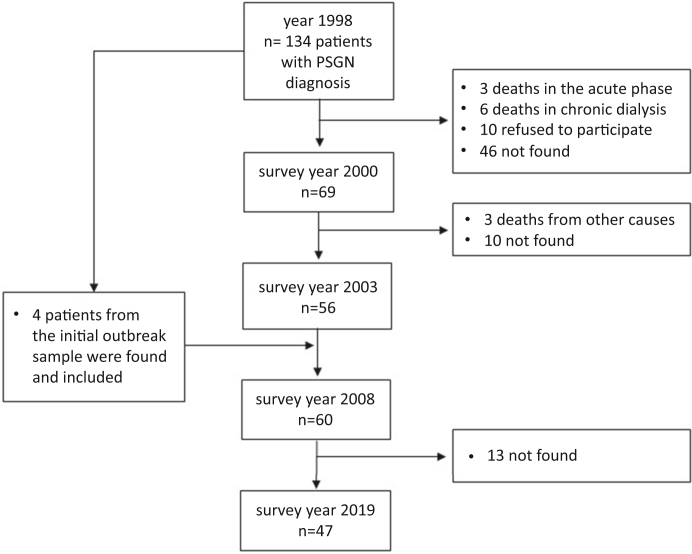
Flow chart of the study population during the follow-up.

Of the 134 confirmed cases of PSGN seen in 1998, 12 died as follows: 3 in the acute phase of illness (the causes of death were sepsis, cerebrovascular accident, and respiratory failure, respectively), 6 died while in a chronic dialysis program before 2 years of follow-up, and 3 with normal renal function died (due to congestive heart failure, myocardial infarction, and stroke) between 2 and 5 years of follow-up. Some cases of the 122 surviving patients could not be located 20 years after the outbreak (*n* = 65) or did not accept to undergo another medical evaluation (*n* = 10), thereby leaving 47 patients for the present study (Figure 1). These participants did not differ significantly from the 87 who were not re-examined regarding gender and mean age at presentation. The mean (range) duration of follow-up of the 47 participants was 20.5 years (20.5–20.6).

Table 1 shows the major sample characteristics as well as clinical and laboratory renal outcomes at 20 years of follow-up. The participants’ mean (SD) age was 56.6 (15.1) years, 12 (25.5%) were 65 years or older, and 62% were women. During the outbreak, 5 (10.6%) patients were 18 years old or younger. Most study participants were nondiabetic (85%) and not obese (94%). Hypertension was present in 34 (72%) patients, 21 (44.7%) had eGFR less than 60 ml/min per 1.73 m^2^, and 8 of 43 (18.6%) patients with measured proteinuria had values greater than 150 mg/g. The whole group’s median (range) of proteinuria was 80 mg/g (30–1450 mg/g). The median (range) proteinuria was 215 mg/g (160–1450 mg/g) in the subgroup with proteinuria greater than 150 mg/g, and 70 mg/g (30–130 mg/g) in the subgroup with proteinuria less than 150 mg/g. Moreover, 25 (53%) patients had CKD (low eGFR and/or proteinuria). Eight patients had proteinuria greater than 150 mg/g; 4 (16%) of these had eGFR lower than 60 ml/min per 1.73 m^2^, and the other 4 (16%) had eGFR higher than 60 ml/min per 1.73 m^2^. The presence of CKD was higher among those older than 65 years than those younger than 65 years (11 of 12, 91.7%; and 14 of 35, 40%, respectively, *P* = 0.002). Two of the 25 (8%) CKD patients were younger than 18 years during the outbreak (12.0 and 13.6 years, respectively). There was no significant association between diabetes, obesity, and degree of proteinuria (Supplementary Results).

**Table 1:** Characteristics of patients with post-streptococcal glomerulonephritis at 20 years of follow-up (*n* = 47)

Parameter	Absolute number or median	Percentage or interquartile range
Age, yrs	55.8	(44.7–66.7)
Gender, female	29	62
Hypertension, yes	34	72.3
Diabetes, yes	7	15.0
Obesity, yes	3	6.5
ACEIs or ARBs use, yes	20	42.5
Laboratory exams		
Hemoglobin, g/dl	13.6	(13.0–14.3)
Hematocrit, %	41.8	(39.5–43.9)
Glycated hemoglobin, %	5.6	(5.2–5.9)
Uric acid, mg/dl	5.3	(4.0–6.6)
Triglycerides, mg/dl	130	(93–159)
Total cholesterol, mg/dl	196	(169–223)
HDLc, mg/dl	59	(50–66)
LDLc, mg/dl	104	(84–135)
Renal parameters		
eGFR, ml/min/1.73 m^2^	61	(49–79)
<30	3	6.4
30–59	18	38.3
≥ 60	26	55.3
Proteinuria, mg/g (*n* = 43)	80	(50–110)
<150	35	81.4
150–500	6	14.0
>500	2	4.7

ACEIs, angiotensin-converting enzyme inhibitor; ARBs, angiotensin receptor blocker; eGFR, estimated glomerular filtration rate; HDLc, high-density lipoprotein cholesterol; LDLc, low-density lipoprotein cholesterol

Table 2 shows the features of patients with and without CKD 20 years after the outbreak. Patients with CKD were older (mean age, 62.9 vs. 50.1 years, *P* = 0.003) and had higher median proteinuria (95 vs. 70 mg/g, *P* = 0.020) than those without CKD. In the logistic regression univariate analysis (Table 3), increasing age was associated with CKD and remained so after adjusting for other confounders in the multivariate model (odds ratio: 1.07; 95% CI: 1.02–1.13; *P* = 0.011). Diabetes mellitus was marginally associated with CKD only in the univariate analysis (odds ratio: 6.63; 95% CI: 0.73–60.21; *P* = 0.093).

**Table 2:** Characteristics of patients with and without chronic kidney disease after 20 years of the post-streptococcal glomerulonephritis outbreak (*n* = 47)

Variable	Without CKD (*n* = 22)	With CKD (*n* = 25)	*P* value
Gender, male/ female	6/16	12/13	0.145
Age, yrs	50.1 (9.9)^a^	62.9 (16.4)^a^	0.003
HbA1C, %	5.4 (0.3)^a^	6.0 (1.4)^a^	0.059
HDLc, mg/dl	60 (11)^a^	59 (12)^a^	0.605
LDLc, mg/dl	119 (8)^a^	105 (9)^a^	0.262
Uric acid, mg/dl	5.3 (1.6)^a^	5.6 (1.9)^a^	0.627
Hemoglobin, g/dl	13.8 (1.1)^a^	13.5 (1.4)^a^	0.434
Proteinuria, mg/g	70 (30–130)^b^	95 (40–1450)^b^	0.020
Systolic BP, mmHg	131 (15)^a^	135 (20)^a^	0.429
Diastolic BP, mmHg	82 (10)^a^	82 (11)^a^	0.968

BP, blood pressure; CKD, chronic kidney disease; HbA1C, glycated hemoglobin; HDLc, high-density lipoprotein cholesterol; LDLc, low-density lipoprotein cholesterol.

CKD = eGFR<60ml/min/1.73 m^2^ and/or proteinuria, protein-to-creatinine ratio >150 mg/g.

a Results expressed as proportions or mean (standard deviation).

b Proteinuria results expressed as median (min-max).

**Table 3:** Univariate and multivariate logistic regression analysis for variables associated with chronic kidney disease after 20 years of the post-streptococcal glomerulonephritis outbreak (*n* = 47)

Variable	Univariate			Multivariate		
	OR	95% CI	*P* value	OR	95% CI	*P* value
Age, yrs	1.07	1.02–1.13	0.007	1.07	1.02–1.13	0.011
Gender, male	2.46	0.72–8.36	0.149	2.46	0.61–9.98	0.206
Systolic BP	1.37	0.42–4.44	0.595			
Diastolic BP	0.68	0.90–2.33	0.540			
Diabetes, yes	6.63	0.73–60.21	0.093	6.55	0.55–77.73	0.137
Obesity, yes	1.00	0.58–1.90	0.879			
Triglycerides, mg/dl	0.99	0.98–1.00	0.203			
VLDLc, mg/dl	0.96	0.92–1.01	0.205			
HDLc, mg/dl	0.98	0.93–1.03	0.597			
LDLc, mg/dl	0.99	0.98–1.00	0.260			
Total cholesterol, mg/dl	0.98	0.98–1.00	0.144			
Hematocrit, %	0.54	0.14–2.08	0.377			
Hemoglobin, g/dl	0.82	0.50–1.33	0.427			
ACEIs or ARBs use, yes	1.61	0.50–5.20	0.422			

ACEIs, angiotensin-converting enzyme inhibitor; ARBs, angiotensin receptor blocker; BP, blood pressure; CI, confidence interval; HDLc, high-density lipoprotein cholesterol; LDLc, low-density lipoprotein cholesterol; OR, odds ratio; VLDLc, very low-density lipoprotein cholesterol.

Table 4 describes the observed mean eGFR values, albuminuria, and hypertension rates at each assessment visit during the follow-up. A substantial increase in mean eGFR values occurred from baseline until 2008 and a pronounced decline in 2019, with a mean eGFR loss of −3.2 ml/min per 1.73 m^2^ per year (95% CI: −3.7 to −2.7) in the last 11 years of follow-up (calculated from 46 patients with measurements on both occasions).

**Table 4:** Descriptive analysis of the observed eGFR, albuminuria, and hypertension rates at each follow-up visit for post-streptococcal glomerulonephritis patients from 1998 to 2019

Year	eGFR ml/min/1.73 m^2^	Albuminuria	Hypertension
1998	58.9±37.9 (47)	NA	70% (134)
2000	88.0±28.4 (36)	34% (65)	42% (64)
2003	84.3±27.0 (30)	22% (51)	30% (56)
2008	100.9±20.5 (46)	12% (60)	45% (60)
2019	65.0±23.0 (47)	19% (43)	72% (47)

NA, not available.

eGFR values are mean±SD. In parenthesis is the number of patients evaluated.

Hypertension is defined as blood pressure ≥140/90 mmHg or the use of antihypertensive drugs.

Albuminuria: values >20 μg/min in the 2000 and 2003 visits, >30 mg/g creatinine in 2008. In 2019 proteinuria was assessed as protein-to-creatinine ratio >150 mg/g.

**Figure 2:**
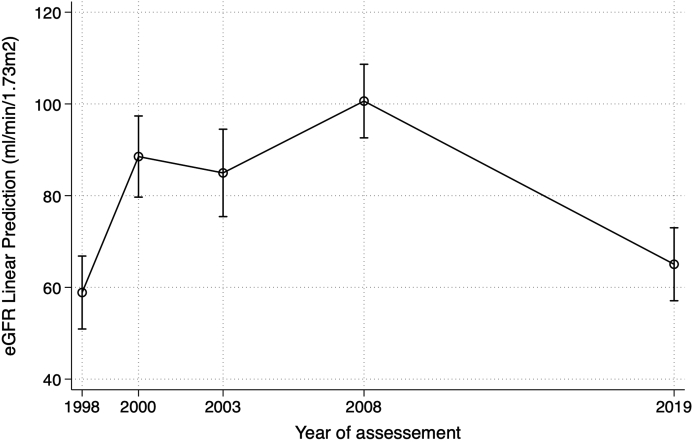
Estimated glomerular filtration rate at follow-up visits in post-streptococcal glomerulonephritis patients during 20-year follow-up using mixed-effects regression analysis. eGFR, estimated glomerular filteration rate. Values are mean and 95% CI. Predicted mean eGFR and number of patients (in parenthesis) at each visit: 59 ml/min per 1.73 m^2^ (*n* = 47), 89 ml/min per 1.73 m^2^ (*n* = 36), 85 ml/min per 1.73 m^2^ (*n* = 30), 101 ml/min per 1.73 m^2^ (*n* = 46), and 65 ml/min per 1.73m^2^ (*n* = 47) in 1998, 2000, 2003, 2008, and 2019, respectively.

Figure 2 shows the predicted mean eGFR values during each visit using the mixed-effects regression model in the cohort of 47 patients. The median number of serum creatinine measures per patient during the follow-up was 3 (minimum of 2, maximum of 4). The predicted mean eGFR values were similar to the observed values and had the same course over time, showing a tendency to increase from 1998 to 2000 and 2008 and a subsequent marked decline in 2019.

**Figure 3:**
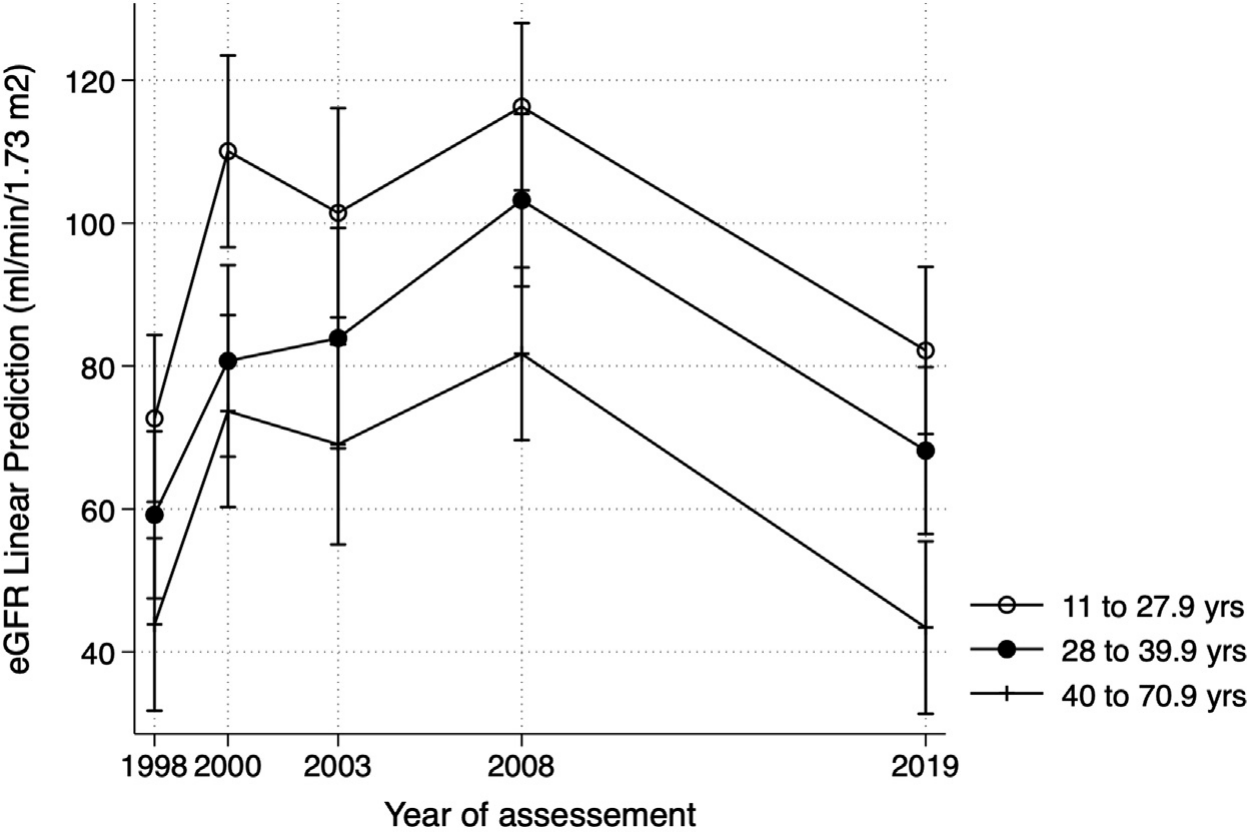
Estimated glomerular filtration rate at follow-up visits by baseline age tertile in post-streptococcal glomerulonephritis patients during 20-year follow-up using mixed-effects regression analysis. Values are mean and 95% CI. Age tertile 1: 11 to 27.9 years; age tertile 2: 28 to 39.9 years; age tertile 3: 40 to 70.9 years.

**Figure 4:**
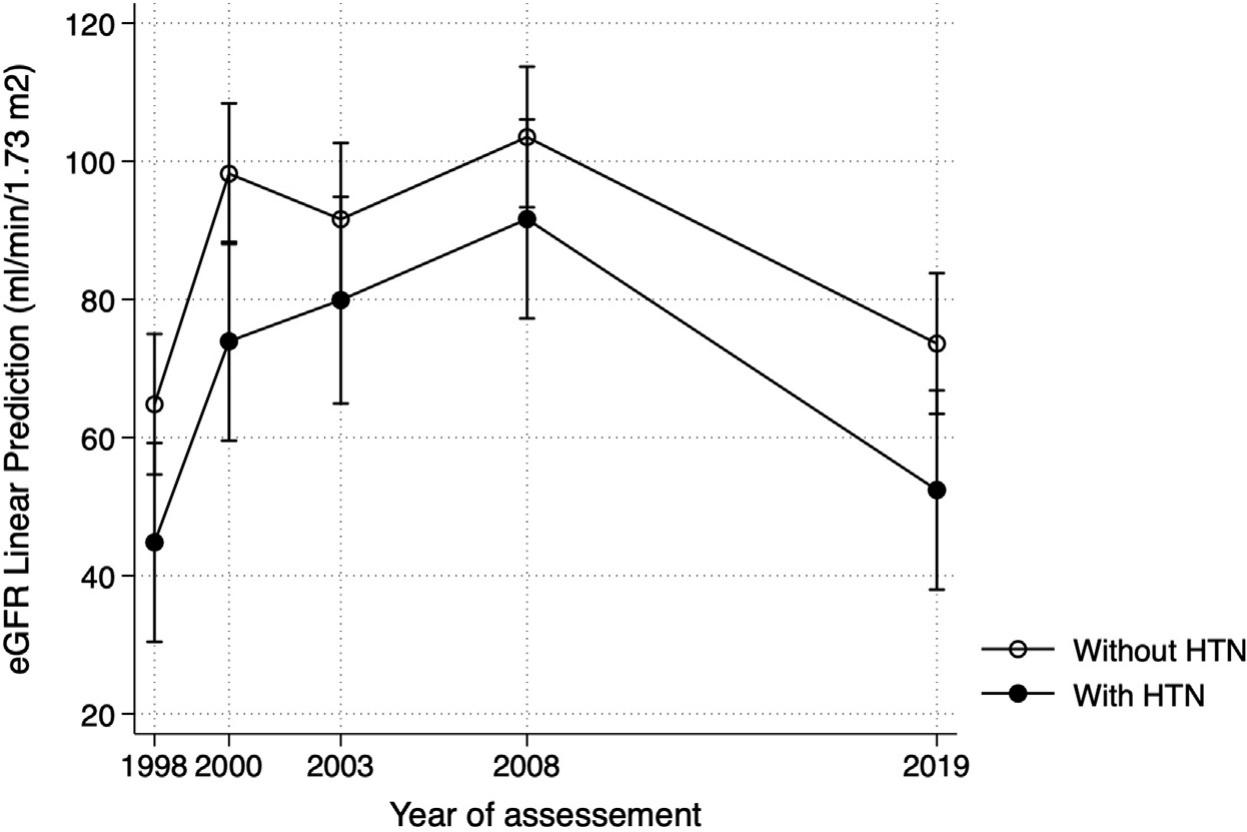
Estimated glomerular filtration rate at follow-up visits by hypertension classification in post-streptococcal glomerulonephritis patients during 20-year follow-up using mixed-effects regression analysis. Values are mean and 95% CI. The presence of hypertension was ascertained at the 2003 visit.

Univariate mixed-effects regression analysis evaluating variables associated with the trajectory of eGFR showed that older age at baseline and hypertension, but not the albuminuria level after 5 years, were associated with a significantly lower eGFR between groups during the follow-up (Table 5). Multivariate analysis confirmed that older age at baseline (coefficient: −1.05 ml/min per 1.73 m^2^ per year; 95% CI: −1.28 to −0.81; *P* < 0.001) and the presence of hypertension 5 years after the outbreak (coefficient: −7.78 ml/min per 1.73 m^2^; 95% CI: −14.67 to −0.88; *P* = 0.027) remained significantly associated with lower eGFR after adjusting for other confounders. Nevertheless, when the models included the interaction terms between these risk factors and follow-up time, these terms were not statistically significant (Supplementary Table S1, S2, and S3). The main effects of age and hypertension on eGFR decline were not different between groups, resulting in curves with similar eGRF trajectories (Figure 3 and 4). In univariate analysis, the mean eGFR in 2019 was not significantly different from the mean value during the outbreak; however, after adjusting for age and hypertension, it was 11/min per 1.73 m^2^ greater at the end of the study (*P* = 0.028). In a further analysis, including data of all 67 available individuals, the results on eGFR trajectories remained similar (Supplementary Table S4).

**Table 5:** Univariate and multivariate analysis by mixed-effect linear regression for variables associated with the estimated glomerular filtration rate trajectory 20 years after the post-streptococcal glomerulonephritis outbreak (*n* = 47)

Variables	Univariate			Multivariate		
	Coefficient (ml/min/1.73 m^2^)	95% CI	*P* value	Coefficient (ml/min/1.73 m^2^)	95% CI	*P* value
Year after the outbreak (1998)^a^						
1998	reference			reference		
2000	29.64	18.72–40.57	<0.001	35.61	25.66–45.55	<0.001
2003	26.07	12.82–39.34	<0.001	32.97	22.46–43.49	<0.001
2008	41.74	31.17–52.31	<0.001	44.42	34.40–54.45	<0.001
2019	6.16	−6.32 to 18.65	0.333	11.18	1.24–21.12	0.028
Gender, male	−4.11	−15.75 to 7.52	0.489			
Baseline age, yrs	−1.02	−1.28 to −0.75	<0.001	−1.05	−1.28 to −0.81	<0.001
Hypertension in 2003, yes	−17.63	−29.68 to −5.58	0.004	−7.78	−14.67 to −0.88	0.027
Albuminuria in 2003, yes	−12.57	−31.34 to 6.20	0.189			

a The coefficient of each survey year is compared with the value at the outbreak year of 1998.

## Discussion

This is the longest follow-up study of adult patients with PSGN. During the 20-year follow-up, patients recovered their eGFR 2 years after the acute phase and continued improving after 10 years; however, there was a marked decline in mean eGFR after 20 years. The mean annual eGFR reduction represented an accelerated drop during the last 11 years. In addition, about half of the sample studied had CKD. Older age at disease onset and the occurrence of hypertension after 5 years were associated with a lower eGFR trajectory.

Long-term follow-up studies to properly evaluate the prognosis after acute PSGN have several obstacles. One such obstacle is the difficulty of asymptomatic individuals adhering to surveillance examinations years after the acute phase of the disease.^14^ Another hurdle is keeping patients’ records and an organized research network group in resource-limited regions to allow their periodic monitoring. The only 2 endemic PSGN studies with an extensive follow-up time, 1 of 12 to 17 years^14^ and the other of 6 to 18 years,^19^ were mainly conducted among children affected by group A beta-hemolytic *Streptococci*. Most GN patients (90%) in the acute phase of the current study outbreak were adults with a mean age of 37 years, and 65% were women.^4^ The most common first symptoms were edema (98%) and high blood pressure (80%). Three of the 10 patients who needed acute hemodialysis (10%) died during the acute phase.^4^

Duca *et al.*^20^ reported the first cases of pharyngitis caused by group C *S zooepidemicus* in Romania; about 90% of the patients were adults and one-third presented GN. As in the few previous reports of human disease due to *S zooepidemicus*,^20^, ^21^, ^22^, ^23^, ^24^, ^25^ the origin of the infection reported in the Minas Gerais outbreak was associated with the consumption of unpasteurized milk,^4^ Patients affected by PSGN due to *S zooepidemicus* are primarily adults and not rarely need dialysis or intensive care unit admission in the early phase of the disease.^21^^,^^23^^,^^25^

Short-term recovery after acute PSGN has been a frequent outcome,^8^^,^^26^ and most studies suggest a benign long-term prognosis of PSGN in children.^27^ Nevertheless, some studies conducted nearly 4 decades ago reported a relevant progression to CKD,^3^^,^^28^ mainly among adults.^1^^,^^3^^,^^12^^,^^29^ In a predominantly adult population with PSGN (*n* = 60), persistent proteinuria, hypertension, or reduction of kidney function were found in 50% of the patients more than 7 years after the acute phase.^1^ Progression to chronic glomerular disease has been found in kidney biopsies conducted about 4 years after the initial event among adult patients.^29^ Nonetheless, misclassification error may partly account for patients’ variable course in long-term PSGN studies because many were based on undocumented infections. Also, nonstandardized and imprecise methods have been used to evaluate kidney function during the follow-up.^8^^,^^26^^,^^27^^,^^30^ Different pathways by which *Streptococci* could initiate and maintain glomerular injury have been postulated since the classical theoretical concept of glomerular trapping of immune complex and complement activation as other structural similarities between streptococcal fractions and kidney structural elements being responsible by a nephritogenic role.^31^ Specific nephritogenic antigens associated with the PSGN caused by *S zooepidemicus* have not been identified.^32^ Whereas a large family of extracellular collagen-like protein genes has been found as a potential target for glomerular-immune complex formation, a genome study of the *S zooepidemicus* of this outbreak ruled out the belief that streptococcal pyrogenic exotoxin B or antibodies reacting with it singularly cause PSGN.^32^

In our study, 72% of the recently examined patients presented with SAH, a remarkably higher percentage than the general population. According to the Brazilian National Health Survey, the largest coverage survey based on self-reporting in Brazil, SAH prevalence is 21.4%.^33^ After adjustment for age and sex and corrected by sensitivity and specificity values of a self-report, SAH prevalence is estimated at 14.5% in the general population (19.5% among men and 11.8% among women).^34^ Prevalence of SAH among the Brazilian age group 40 to 59 years is 13% (95% CI: 11.3–14.7%), 20.6% among men, and 9.3% among women.^34^ Although the exact SAH prevalence in the general population of the cities involved in the outbreak is unknown, the high prevalence of SAH found among the re-examined patients was more than twice that found in a control group matched by sex and age for the same patients reported 10 years after the outbreak.^3^ Another comorbidity that could be implicated in our results is diabetes. The prevalence of diabetes in our sample was similar to that of the general Brazilian populational adjusted by age group.^35^ Although diabetes was marginally associated with a higher risk of CKD in the univariate analysis, the relatively small sample size hindered further assessment of this condition.

Decline of glomerular filtration rate was observed after the fourth decade of life in the general population,^36^ and the loss of functioning nephrons would be responsible for an annual reduction in glomerular filtration rate of 0.75 ml/min per 1.73 m^2^.^36^, ^37^, ^38^ Hypertension has been associated with eGFR decline over time in large observational studies in the general population.^39^, ^40^, ^41^ At the end of this follow-up study, the mean eGFR was 11 ml/min per 1.73 m^2^ above the mean value observed during the outbreak, and half of the studied sample fulfilled CKD criteria. Our sample’s mean eGFR decline rate during the last 11 years of follow-up was notably greater than expected in the general population and among hypertensive patients (−0.85 to −1.50 ml/min/1.73 m^2^ per year).^38^^,^^42^ A large prospective cohort with more than 14,000 employees (median age, 51 years) in the public sector from 6 Brazilian capitals has found a much lower prevalence of CKD (eGFR <60ml/min per 1.73 m^2^ in 4.8% and albumin-to-creatinine ratio >300mg/g in 0.6% of participants) than in our group.^43^ The prevalence of SAH in this Brazilian cohort study was also lower (36%) than in our sample. Other Brazilian populational studies have found a prevalence of CKD (eGFR <60ml/min per 1.73 m^2^) in the general adult population between 6.7%^44^ and 8.7%.^45^ Increased proteinuria was detected in 19% of our patients, and 4 of them (9.3%) had proteinuria without reduced eGFR. This proteinuria rate is clearly greater than that reported for the general population in the literature (about 7%).^46^ Hypertension and albuminuria levels have been considered strong risk factors for decline in glomerular filtration rate.^38^ We found an association between hypertension at 5 years and a lower eGFR trajectory, although hypertension was not associated with faster eGFR decline. Albuminuria did not relate to eGFR, which may reflect the low levels of albuminuria our patients presented and the low power of our study to detect this effect. Nevertheless, considering the increased prevalence of abnormal levels of proteinuria in previous reassessments, it is not possible to rule out a progressive nonimmunological glomerular lesion after the acute outbreak phase as part of the progression to CKD.

Aging has been linked with a higher risk of CKD.^31^ In a multivariate analysis, we observed that older age at presentation was related to CKD after 20 years of the outbreak; at the last evaluation, 92% of the patients 65 years or older had CKD. Moreover, older age was significantly associated with a lower eGFR trajectory in the mixed-effects regression analysis. Baldwin *et al.*^1^ suggested that younger patients had better prognoses than older people with PSGN in long-term follow-up (2 to 15 years). It is conceivable that older patients have lost a substantial proportion of their nephrons during the acute phase of GN. Over the ensuing 20 years, the reduced nephron mass may have decreased the eGFR due to glomerular hyperperfusion or hyperfiltration injury.

The present study has some limitations. It is not possible to rule out a selection bias considering the patients who could not be included in this reassessment. It was difficult to find and contact the patients due to the very long follow-up since the outbreak and some individuals’ residence city changed. Most nonparticipants were not located, and just a few contacted individuals refused to participate during the whole follow-up. We did not observe significant differences between the participants and those not evaluated regarding sex and age. In addition, the limited sample size may have reduced the statistical power of some analyses. We could not obtain age and sex-matched community controls in this study. Kidney biopsies could have improved the comprehension of the CKD evolution. Nevertheless, because it was a disease of already recognized etiology and many patients were asymptomatic, ethical aspects hampered the procedure.

Long-term follow-up studies after PSGN are rare. This report is the longest follow-up study of epidemic PSGN mainly due to *S zooepidemicus*, in the literature,. The participant’s cohort was followed by some of the same authors who described the outbreak more than 20 years ago. Univariate and multivariate analyses were performed using several clinical and laboratory parameters in the previous evaluation and eGFR measurements during the follow-up visits.

In conclusion, after a mean follow-up of 20 years, more than half of the patients with PSGN due to *S zooepidemicus* had CKD, and 72% had hypertension. Mean eGFR declined markedly 2 decades after the outbreak. The present findings emphasize the importance of long-term continuous follow-up after PSGN in adults, especially older adults in the acute phase of the disease and those with hypertension.

## Disclosure

All the authors declared no competing interests.

## Supplementary Material

Supplementary File (PDF)

**Supplementary Results.**

**Table S1.** Mixed-effect linear regression analysis for the estimated glomerular filtration rate trajectory after the post-streptococcal glomerulonephritis outbreak, testing the effect of age and the interaction terms with time (*n* = 47).

**Table S2.** Mixed-effect linear regression analysis for the estimated glomerular filtration rate trajectory after the post-streptococcal glomerulonephritis outbreak, testing the effect of hypertension and the interaction terms with time (*n* = 47).

**Table S3.** Mixed-effect linear regression analysis for the estimated glomerular filtration rate trajectory after the post-streptococcal glomerulonephritis outbreak, testing the effect of gender and the interaction terms with time (*n* = 47).

**Table S4.** Univariate mixed-effect linear regression analysis for the estimated glomerular filtration rate trajectory during the follow-up after the post-streptococcal glomerulonephritis outbreak, including all available subjects (*n* = 67).

**STROBE statement.**
