# Supplementary material for: Twenty-year Follow-up of Patients With Epidemic Glomerulonephritis due to Streptococcus zooepidemicus in Brazil
Source: Kidney Int Rep. 2022 Jun 30;7(9):2029–38. doi: 10.1016/j.ekir.2022.06.011 (PMC9458988; doi:10.1016/j.ekir.2022.06.011)
Supplement: Supplementary File (PDF) [file mmc1.pdf]

Supplementary Material

Table S1. Mixed-effect linear regression analysis for the eGFR trajectory after the PSGN outbreak, testing the effect of age and the interaction terms with time (n=47).

Variable	Coefficient (ml/min./1.73m^2)	95% CI	*p* value
Time			
1998	reference		
2000	37.39	20.73 to 54.05	<0.001
2003	28.77	11.11 to 46.43	=0.001
2008	43.63	28.33 to 58.94	<0.001
2019	9.51	-5.79 to 24.82	0.223
Age tertile			
1	reference		
2	-13.49	-30.02 to 3.04	0.110
3	-28.83	-45.64 to -12.03	=0.001
Time*age tertile			
2000*age tertile 2	-15.85	-39.42 to 7.71	0.187
2000*age tertile 3	-7.55	-31.26 to 16.17	0.533
2003*age tertile 2	-4.05	-29.48 to 21.39	0.755
2003*age tertile 3	-3.57	-28.31 to 21.17	0.777
2008*age tertile 2	0.40	-21.44 to 22.25	0.971
2008*age tertile 3	-5.75	-27.75 to 16.25	0.608
2019*age tertile 2	-0.52	-22.16 to 21.13	0.963
2019*age tertile 3	-9.95	-31.95 to 12.06	0.376

Age tertile 1: 11 to 27.9 years; age tertile 2: 28 to 39.9 years; age tertile 3: 40 to 70.9 years

Table S2. Mixed-effect linear regression analysis for the eGFR trajectory after the PSGN outbreak, testing the effect of hypertension and the interaction terms with time (n=47).

Variable	Coefficient ($ml/min/1.73m^2$)	95% CI	*p* value
Time			
1998	reference		
2000	33.40	21.58 to 45.21	<0.001
2003	26.81	14.25 to 39.37	<0.001
2008	38.70	26.88 to 50.52	<0.001
2019	8.79	-3.03 to 20.61	0.145
Hypertension			
No	reference		
Yes	-19.99	-37.63 to -2.35	0.026
Time*hypertension			
2000*hypertension	-4.28	-24.75 to 16.19	0.682
2003*hypertension	8.28	-13.01 to 29.57	0.446
2008*hypertension	8.12	-12.35 to 28.59	0.437
2019*hypertension	-1.21	-21.68 to 19.26	0.908

Table S3. Mixed-effect linear regression analysis for the eGFR trajectory after the PSGN outbreak, testing the effect of gender and the interaction terms with time (n=47).

Variable	Coefficient (ml/min./1.73m^2)	95% CI	*p* value
Time			
1998	reference		
2000	29.82	21.28 to 38.36	<0.001
2003	25.20	16.05 to 34.35	<0.001
2008	39.13	31.14 to 47.11	<0.001
2019	5.60	-3.02 to 14.21	0.203
Gender			
Female	reference		
Male	0.47	-15.82 to 16.75	0.955
Time*gender			
2000*gender (male)	-7.71	-27.99 to 12.57	0.456
2003*gender (male)	-2.80	-23.91 to 18.31	0.800
2008*gender (male)	-5.54	-24.10 to 13.02	0.559
2019*gender (male)	-9.99	-28.47 to 8.50	0.290

Table S4. Univariate mixed-effect linear regression analysis for the eGFR trajectory during the follow-up after the post-streptococcal glomerulonephritis outbreak, including all available subjects (n=67).

Variable	Coefficient (ml/min./1.73m^2)	95% CI	*p* value
Year after the outbreak (1998)*			
1998	reference		
2000	29.82	21.28 to 38.36	<0.001
2003	25.20	16.05 to 34.35	<0.001
2008	39.13	31.14 to 47.11	<0.001
2019	5.60	-3.02 to 14.21	0.203

Supplementary Results.

-Tests of association between diabetes, obesity, and proteinuria.

Proteinuria (protein to creatinine ratio >150 mg/g) was present in 3/6 (50%) and 5/37 (13.5%) of diabetic and non-diabetic patients, respectively (p=0.067).

Proteinuria (protein to creatinine ratio >150 mg/g) was present in 1/3 (33.3%) and 7/39 (18%) of obese and non-obese patients, respectively (p=0.479).

Modified STROBE Statement—checklist of items that should be included in reports of observational studies (Cohort/Cross-sectional and case-control studies)

	Item No	Recommendation
**Title and abstract**	1	(a) Indicate the study's design with a commonly used term in the title or the abstract<br><br>Yes, Pg 1 and pg 2
		(*b*) Provide in the abstract an informative and balanced summary of what was done and what was found<br><br>Yes, Pg 2
**Introduction**		
Background/rationale	2	Explain the scientific background and rationale for the investigation being reported<br><br>Yes, Pg 4 and pg 5
Objectives	3	State specific objectives, including any prespecified hypotheses<br><br>Yes, Pg 5
**Methods**		
Study design	4	Present key elements of study design early in the paper<br><br>Yes, Pg 5 3rd parag
Setting	5	Describe the setting, locations, and relevant dates, including periods of recruitment, exposure, follow-up, and data collection<br><br>Yes, Pgs 5-7
Participants	6	*(a) Cohort study*—Give the eligibility criteria, and the sources and methods of selection of participants. Describe methods of follow-up<br><br>Yes, Pg 5 3rd parag., pg 7<br><br>*Case-control study*—Give the eligibility criteria, and the sources and methods of case ascertainment and control selection. Give the rationale for the choice of cases and controls<br><br>*Cross-sectional study*—Give the eligibility criteria, and the sources and methods of selection of participants
Variables	7	Clearly define all outcomes, exposures, predictors, potential confounders, and effect modifiers. Give diagnostic criteria, if applicable

Data sources/ measurement	8*	For each variable of interest, give sources of data and details of methods of assessment (measurement).<br><br>Yes, Pg 5 – pg 9
Bias	9	Describe any efforts to address potential sources of bias<br><br>Yes, Pg 5 and pg 6
Study size	10	Explain how the study size was arrived at (if applicable)<br><br>Yes, Pgs 6,8,9
Quantitative variables	11	Explain how quantitative variables were handled in the analyses. If applicable, describe which groupings were chosen and why<br><br>Yes, Pgs 7-9
Statistical methods	12	*(a)* Describe all statistical methods, including those used to control for confounding<br><br>Yes, Pgs 8,9
		(*b*) Describe any methods used to examine subgroups and interactions<br><br>Yes, Pgs 8,9
		(*c*) Explain how missing data were addressed<br><br>Yes, Pg 9 3rd parag., pg 10
		(*d*) *Cohort study*—If applicable, explain how loss to follow-up was addressed<br><br>Yes, Pg 9 3rd parag., pg 10<br><br>*Case-control study*—If applicable, explain how matching of cases and controls was addressed<br><br>*Cross-sectional study*—If applicable, describe analytical methods taking account of sampling strategy
		(*e*) Describe any sensitivity analyses<br><br>Yes, Pg 12, 1st parag.
**Results**		
Participants	13*	(a) Report numbers of individuals at each stage of study—eg numbers potentially eligible, examined for eligibility, confirmed eligible, included in the study, completing follow-up, and analyzed

		Yes, Pg 9 3rd parag, Fig 1
		(c) **Use of a flow diagram**<br>Yes, **Figure 1**
Descriptive data	14*	(a) Give characteristics of study participants (eg demographic, clinical, social) and information on exposures and potential confounders<br>Yes, Pg 9 and pg 10
		(b) Indicate number of participants with missing data for each variable of interest<br>Yes, Pg 10 2nd parag, Pg 11 3rd parag, Table 4 and Fig 2 footnotes
		(c) *Cohort study*—Summarise follow-up time (eg, average and total amount)<br>Yes, Pg 10 1st parag
Outcome data	15*	*Cohort study*—Report numbers of outcome events or summary measures over time<br>Yes, Pg 10,11,12 1st parag, Tables 1,4
		Case-control study—Report numbers in each exposure category, or summary measures of exposure
		Cross-sectional study—Report numbers of outcome events or summary measures
Main results	16	(*a*) Give unadjusted estimates and, if applicable, confounder-adjusted estimates and their precision (eg, 95% confidence interval). Make clear which confounders were adjusted for and why they were included<br>Yes, Pgs 10,11,12 1st parag, Tables 2,3,5, supplementary Table S1-S3
Other analyses	17	Report other analyses done—eg analyses of subgroups and interactions, and sensitivity analyses<br>Yes, Pg 11 2nd parag, pg 12 1st. parag.Table 2, Tables S1, S2, S3
**Discussion**		
Key results	18	Summarise key results with reference to study objectives<br>Yes, Pg 12 2nd parag

Limitations	19	Discuss limitations of the study, taking into account sources of potential bias or imprecision. Discuss both direction and magnitude of any potential bias<br><br>Yes, Pg 16 2nd parag, pg 17
Interpretation	20	Give a cautious overall interpretation of results considering objectives, limitations, multiplicity of analyses, results from similar studies, and other relevant evidence<br><br>Yes, Pg 16 2nd and 3rd parag., Pg 17 2nd parag.
Generalisability	21	Discuss the generalisability (external validity) of the study results<br><br>Yes, Pg 16 2nd and 3rd parag., Pg 17 2nd parag.

*Give information separately for cases and controls in case-control studies and, if applicable, for exposed and unexposed groups in cohort and cross-sectional studies.

Note: An Explanation and Elaboration article discusses each checklist item and gives methodological background and published examples of transparent reporting. The STROBE checklist is best used in conjunction with this article (freely available on the Web sites of PLoS Medicine at http://www.plosmedicine.org/, Annals of Internal Medicine at http://www.annals.org/, and Epidemiology at http://www.epidem.com/). Information on the STROBE Initiative is available at www.strobe-statement.org.
